# Huangkui Capsule in Combination with Leflunomide Improves Immunoglobulin A Nephropathy by Inhibiting the TGF-β1/Smad3 Signaling Pathway

**DOI:** 10.6061/clinics/2021/e2904

**Published:** 2021-11-25

**Authors:** Shuwen Pei, Yan Li

**Affiliations:** I Department of Nephrology, Harbin First Hospital, Harbin, Heilongjiang 15000, China.; II Intensive Care Unit, Harbin First Hospital, Harbin, Heilongjiang 15000, China.

**Keywords:** IgA Nephropathy, Huangkui Capsules, Leflunomide, Th22 Cells, TGF-β1/Smad3 Signaling Pathway

## Abstract

**OBJECTIVES:**

To investigate the efficacy and potential molecular mechanism of Huangkui capsule in combination with leflunomide (HKL) for the treatment of immunoglobulin A nephropathy (IgAN)

**METHODS:**

IgAN rat models were constructed by treating rats with bovine serum albumin, lipopolysaccharide, and tetrachloromethane. Th22 cells were isolated from the blood samples of patients with IgAN using a CD4+ T cell isolation kit. The expression levels of the components of the TGF-β1/Smad3 signaling pathway, namely, TGF-β1, Smad2, Smad3, Smad4, and Smad7, were detected using quantitative reverse transcription polymerase chain reaction. Cell proliferation was determined using the MTT assay, cell viability was determined using the WST 1 method, and the chemotaxis of Th22 cells was observed using the wound healing assay. Changes in the histology of the kidney tissues were analyzed using hematoxylin and eosin staining.

**RESULTS:**

Compared with IgAN rats, the rats subjected to HKL treatment showed good improvement in kidney injuries, and the combined drug treatment performed much better than the single-drug treatment. In addition, following HKL treatment, the viability, proliferation, and chemotaxis of Th22 cells dramatically decreased (*p<0.05, **p<0.01, and ***p<0.001). In addition, CCL20, CCL22, and CCL27 levels decreased and the expression of the key components of the TGF-β1/Smad3 signaling pathway was downregulated in IgAN rats and Th22 cells (*p<0.05, ***p<0.001).

**CONCLUSIONS:**

By targeting the TGF-β1/Smad3 signaling pathway, HKL treatment can improve kidney injury in IgAN rats as well as the excessive proliferation and metastasis of Th22 cells.

## INTRODUCTION

Primary immunoglobulin A nephropathy (IgAN) is one of the most common glomerular diseases in children and teenagers and is mainly characterized by proteinuria, filtration membrane injury, and glomerulonephritis (1,2). In China, the development of IgAN is the major cause of end-stage kidney diseases (3-5). Delayed or inappropriate treatment of patients with IgAN in the early stage resulted in permanent disability in approximately 30% of these patients (4,6).

Currently, there is insufficient evidence regarding the pathogenesis of IgAN, which prevents the development of efficient strategies for the treatment of IgAN. However, according to current research, immunological factors and signaling pathways are related to the development of IgAN (7-9). For example, the level of transforming growth factor β1 (TGF-β1) in glomeruli is positively correlated to matrix accumulation (10). TGF-β1 is a key regulatory factor in liver fibrosis and inflammation, as well as in the growth, apoptosis, and differentiation of cells (11). Its downstream signal transduction is mediated by Smad2 and Smad3, which are phosphorylated by the heteromultimer of the TGF-β1 receptor at the SSXS of the C-terminal (12), which binds to Smad4 and translocates into the nucleus for transcriptional regulation (12).

The Huangkui capsule (HKC) and leflunomide (LEF) are drugs that are frequently used for the treatment of chronic inflammation. HKC is a capsule comprising the effective constituents extracted from *Abelmoschus manihot* (L.) medic. It has been approved by the National Medical Products Administration of the People’s Republic of China (Z19990040) for the treatment of chronic nephritis (13). Existing evidence has revealed that the major constituents of HKC are isoquercitrin (C_21_N_20_O_12_), myricetin (C_15_N_10_O_8_), quercetin-3-O-D-glucoside (C_21_N_20_O_12_), quercetin (C_15_N_10_O_7_), hyperoside (C_21_N_20_O_12_), and gossypetin (C_15_N_10_O_8_) (14). HKC can ameliorate nephritis, nephrotic syndrome, anaphylactoid purpura nephritis, IgAN, membranous nephropathy, and diabetic nephropathy (14-16). LEF, an immunosuppressant, exhibits promising efficacy in some immune-related diseases and has frequently been used in the clinical treatment of IgAN.

Therefore, the present study aimed to investigate the efficacy and pathogenesis of HKC in combination with LEF (HKL) for the treatment of IgAN and to determine its effects on the TGF-β1/Smad3 signaling pathway.

## MATERIALS AND METHODS

### Ethical approval

The procedures of this study were approved by the Ethical Board of Harbin First Hospital, and all participants provided written informed consent prior to enrollment. The study protocols involving animal experiments were reviewed and approved by the Animal Care and Ethics Committee.

### Participants

We recruited a total of 33 participants: 15 healthy participants (control group) and 18 patients with IgAN (IgAN group). The criteria for inclusion of patients with IgAN were as follows: (1) age >16 years, (2) diagnosis of IgAN via kidney biopsy, and (3) absence of any other immune-related diseases or complications. The criteria for inclusion of healthy participants were as follows: (1) absence of kidney disease and (2) presence of other immune diseases or known infections.

### Quantitative reverse transcription polymerase chain reaction (qRT-PCR)

The expression levels of TGF-β1, Smad2, Smad3, Smad4, and Smad7 in the TGF-β1/Smad3 signaling pathway were measured using qRT-PCR. The TRIzol reagent (Invitrogen, Carlsbad, CA, USA) was used to isolate total RNA from the cells according to the manufacturer’s instruction, and RNA concentration and purity were evaluated using a NanoDrop spectrophotometer (NanoDrop Technologies, Wilmington, DE, USA). Complimentary DNA was prepared via reverse transcription using the iScript kit (Bio-Rad, Hercules, CA, USA). qRT-PCR was performed using the Power SYBR Green qRT-PCR kit (Life Technologies, Shanghai, China) with fluorescent bases for amplification using the TaqMan Master Mix (Applied Biosystems, CA, USA). Quantitative analysis data were collected using the ABI 7500 Fast System (Applied Biosystems) and 2^−ΔΔCt^ method. The results were normalized to glyceraldehyde-3-phosphate dehydrogenase level.

### Construction of IgAN rat models

A total of 32 healthy, male, specific pathogen-free Sprague Dawley rats aged between 6 and 7 weeks and weighing 200±20g were provided by the Laboratory Animal Center of Guangdong and were fed in a specific pathogen-free environment. Tests for the presence of urinary proteins showed negative results. In addition to the eight rats in the control group, the remaining rats were treated with bovine serum albumin (BSA), lipopolysaccharide (LPS), and tetrachloromethane (CCL_4_) to construct IgAN rat models according to previously described methods (17-20). After 1 week of adaptation, BSA (600 mg/kg) was administered orally every other day. All rats were injected with 0.05 mg of LPS via the caudal veins in the sixth and eighth weeks, followed by injection with 0.1 mL of CCl_4_ every week for 12 weeks. We randomly grouped the IgAN rat models into four groups: the model group (IgAN group), HKC group (2 g/kg/day), LEF group (5 mg/kg/day), and HKL group. The control and model groups were orally administered normal saline (10 mL/kg/day) once daily for 4 weeks.

### Measurement of protein levels and red blood cell counts in urine

At the end of the 1^st^, 6^th^, 9^th^, 12^th^, 13^th^, 14^th^, 15^th^, and 16^th^ weeks, a specially designed metabolimeter was used to collect 24-h urine samples, during which time rats had limited access to food but had unlimited access to water. The levels of urinary proteins were determined via colorimetry using Coomassie Brilliant Blue. Thereafter, hematuresis was observed using a light microscope, and the count of red blood cells was averaged. Samples with ≥3 red blood cells in each field under the microscope were considered positive for hematuresis.

### Hematoxylin & eosin (HE) staining and pathological criteria

The kidney and liver tissues of rats were collected simultaneously after IgAN induction and the corresponding treatment. The kidney tissues of rats were fixed in 4% paraformaldehyde, embedded in paraffin, and cut into 5-μm-thick sections, which were then stained with HE for histological and collagen analyses (21). Semiquantitative evaluation of HE-stained sections was performed, and kidney injury was graded from 0 to 4 as follows: 0=normal tissue; 1=changes affecting *<*25% of the sample; 2=changes affecting 25%-50% of the sample; 3=changes affecting 50%-75% of the sample; and 4=changes affecting >75% of the sample (22). The updated Oxford Classification (MEST-C) was used to score and judge the experimental results, which were independently diagnosed and scored by two pathologists (23).

### Immunohistochemical (IHC) analysis

The kidney sections (4 mm thickness) were fixed with paraformaldehyde, mounted on slides, dewaxed, and hydrated. The slides were placed in 10 mM sodium citrate buffer (pH 6) for 2 min and then cooled for 30 min. After incubation in 3% hydrogen peroxide for 15 min, the sections were blocked with goat serum for 30 min and then incubated overnight with primary antibodies at 4°C. After washing with PBST buffer solution, the cells were incubated with HRP combined with anti-rabbit and anti-mouse IgG for 30 min. A diaminobenzidine tetrahydrochloride solution was used as a chromogenic agent, and hematoxylin was used for redyeing to determine the location of the peroxidase complex.

### Evaluation of the chemotaxis of Th22 cells

CD4+ T cells were isolated and purified from patients with IgAN and healthy participants using a CD4+ T cell isolation kit (Miltenyi Biotec, Bergisch Gladbach, Germany). CD4+ T cells isolated from patients with IgAN were divided into four groups: IgAN group, HKC group (0.5 g/mL of HKC), LEF group (1 mg/mL), and HKL group. Those isolated from healthy participants were considered the control group. These cells were added to the upper chambers of 24-well plates supplemented with RPMI-1640 medium containing 0.5% fetal bovine serum in a final volume of 100 μL, whereas in the lower chamber, 600 μL of the supernatant of HMC (provided by the Advanced Research Center of Central South University) was added. Then, Transwell plates were preserved at 37°C in 5% CO_2_ for 5h. Next, Th22 cells in the lower chambers were analyzed using flow cytometry and classified by their chemotactic index (chemotactic index=quantity of migrated Th22 cells in each group/quantity of migrated Th22 cells in the control group). Migrated cells were fixed with 95% ethanol and stained with 0.1% crystal violet, followed by cell counting using a microscope (Olympus, Tokyo, Japan). The levels of CCL20, CCL22, and CCL27 were determined using ELISA kits (R&D, Minneapolis, MN, USA).

### MTT and WST-1 assays

According to the standard protocol, Th22 cells were seeded into a 96-well plate at a density of 5000 cells/well and cell proliferation was measured using the CCK8 Kit (7Sea Biotech, Shanghai, China) according to the manufacturer’s instructions. Cell viability was evaluated using the WST-1 assay (Roche Diagnostics GmbH, Mannheim, Germany) (24).

### Statistical analysis

Statistical analysis was performed using GraphPad Prism 8.0 (GraphPad, La Jolla, CA, USA). All data are expressed as mean±SD. Differences were considered statistically significant at *p<*0.05.

## RESULTS

### Key components of the TGF-β1/Smad3 signaling pathway are upregulated in patients with IgAN

**Figure 1:**
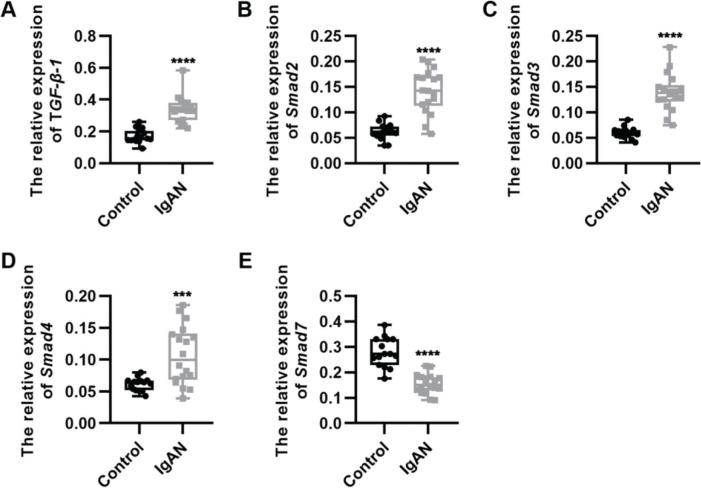
Key components of the TGF-β1/Smad3 signaling pathway are upregulated in patients with IgAN. (A-D) Expression levels of TGF-β1 (A), Smad2 (B), Smad3 (C), and Smad4 (D) are upregulated in patients with IgAN. (E) The expression level of Smad7 is downregulated in patients with IgAN. IgAN: Immunoglobulin A nephropathy, ***p<0.0001 and ****p<0.00001, Mann-Whitney *U* test (A), unpaired Student’s *t*-test (B-E).

We performed qRT-PCR to determine the expression levels of the key components of the TGF-β1/Smad3 signaling pathway in patients with IgAN. We observed that the expression levels of TGF-β1, Smad2, Smad3, and Smad4 were upregulated, whereas those of Smad7, which inhibits the phosphorylation of Smad2 and Smad3, were downregulated (Figure 1A-E, ***p<0.0001 and ****p<0.00001).

### HKL reduces levels of urinary proteins and ameliorates glomerular injury in rats with IgAN

**Figure 2:**
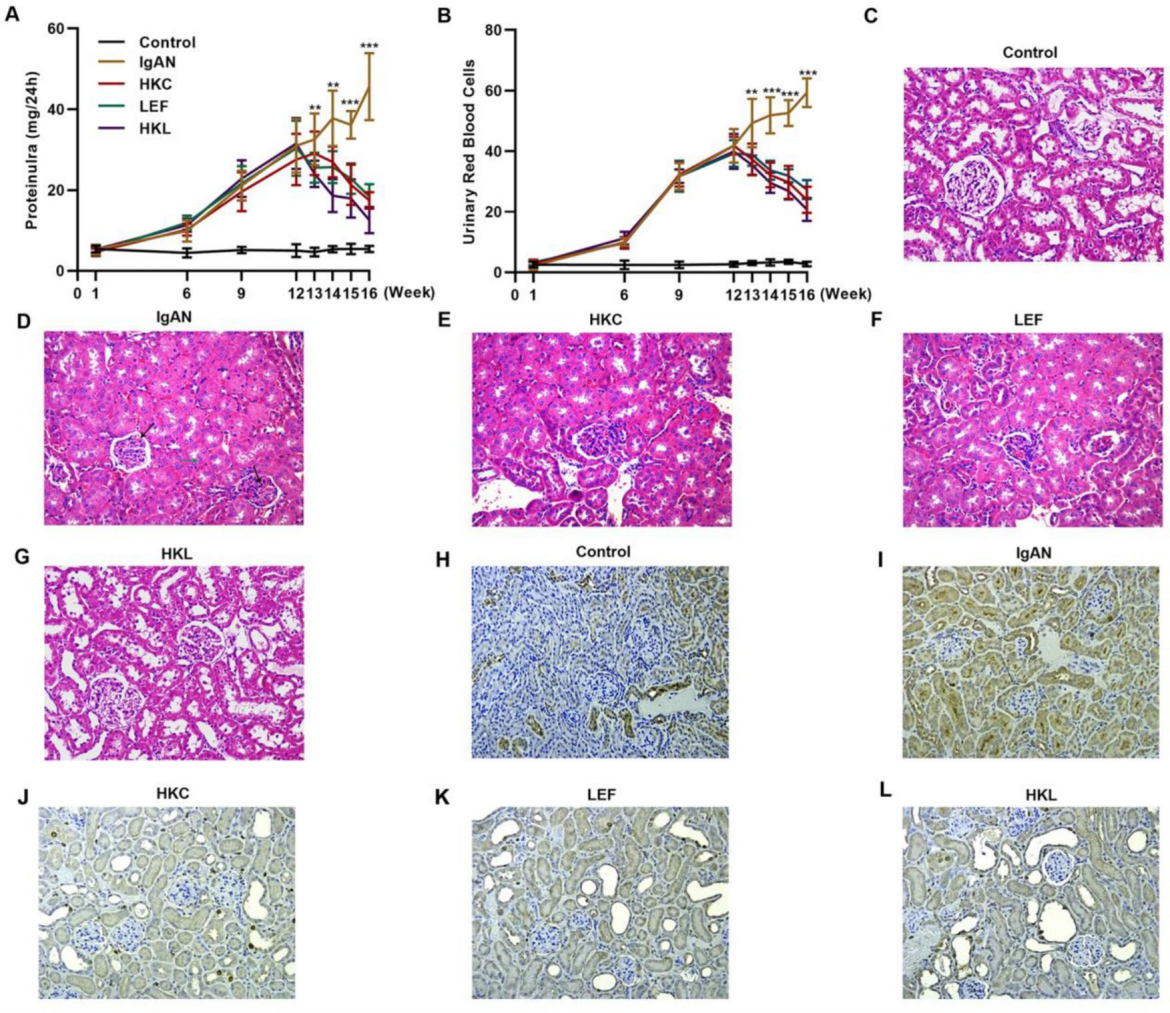
HKL reduces the levels of urinary proteins and ameliorates glomerular injury in IgAN rats. (A-B) HKL reduces urinary protein levels (A) and red blood cell counts (B) in IgAN rats. (C-G) Pathological analysis of the kidney tissues of the rats in each group (black arrows indicate proliferation of glomerular mesangial cells and green arrows indicate infiltration of inflammatory cells in the tubulointerstitial area). (H-L) IHC of kidney tissues of rats in each group. HKC: Huangkui capsule, LEF: leflunomide, HKL: HKC in combination with LEF, IgAN: Immunoglobulin A nephropathy, **p<0.001 and ***p<0.0001, Mann-Whitney *U* test.

**Figure S1:**
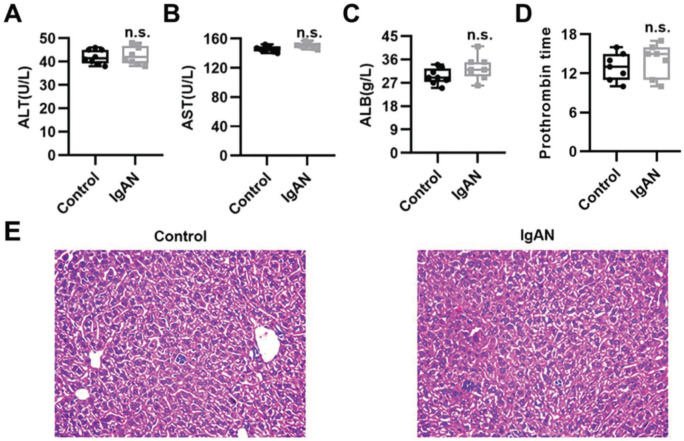
Construction of IgAN rat model had no impact on the liver. (A-D) The IgAN rats constructed by CCl4 method showed no obvious abnormality of ALT (A), AST (B), ALB (C) and PT indicators (D). (E) The results of HE staining. ALT: alanine aminotransferase, AST: aspartate aminotransferase, ALB: albumin, IgAN: Immunoglobulin A nephropathy.

To validate the efficacy of HKL on IgAN, we constructed an IgAN rat model that was later treated with HKL for 4 weeks, followed by measurement of urinary protein levels. First, eight rats that received continuous injections of 0.1 mL CCl_4_ for 12 weeks were randomly selected, and alanine aminotransferase (ALT) level, aspartate aminotransferase (AST) level, albumin (ALB) level, and prothrombin time (PT) were detected. The results showed that IgAN rats constructed by the CCl_4_ method showed no obvious abnormalities in ALT, AST, ALB, and PT indicators (Figure S1A-D). To further verify that the livers of IgAN rats were not damaged, the kidney structure of IgAN rats was anatomically examined, and the liver status of IgAN rats was observed by HE staining. The results of HE staining showed that the liver lobules of IgAN rats were intact, the morphology of liver cells was intact, the normal nucleus was not enlarged, the boundary of hepatic cords was clear, and no obvious abnormality was found in the sinusoidal system (Figure S1E). These results indicate that our construction method was specific to kidney injury and had no significant impact on the liver. IgAN rats showed increased urinary protein levels and red blood cell counts, which were higher than those in the control group (Figure 2A-B, **p<0.001 and ***p<0.0001). However, after 1 week of HKL treatment, a significant decrease was observed in these indicators in IgAN rats (Figure 2A-B, **p<0.001 and ***p<0.0001). Although decreases were detected in IgAN rats after a single administration of HKC or LEF for 2 weeks, these decreases were significantly different from those in the HKL group (Figure 2A-B, **p<0.001 and ***p<0.0001). To further validate the efficacy of HKL in IgAN rats, we evaluated pathological kidney injury in IgAN rats using HE staining. As indicated in Figure 2C-F, the control group had no significant anomalies and presented with closely organized intercapillary cells and renal tubules (kidney injury grade=0). IgAN rats had obvious pathological kidney injuries and exhibited proliferation of glomerular mesangial cells, increased mesangial matrices, significant tubule dilation, vacuolization of tubular epithelial cells, and infiltration of the tubulointerstitial area by inflammatory cells (kidney injury grade=4). However, after HKL treatment, the proliferation of mesangial cells decreased, the mesangial matrices returned to normal, vacuolization of renal tubular epithelial cells decreased, and level of inflammatory cells in the tubulointerstitial area decreased (kidney injury grade=0). Single treatment with HKC or LEF for 2 weeks can somehow improve kidney injury, but it still lacks the efficacy of HKL (kidney injury grade=1). Subsequently, IHC was used to observe the IgA deposition in the glomeruli. The results showed that there was no IgA deposition in the control group, whereas there was more IgA deposition in the IgAN group. Single treatment with HKC or LEF reduced IgA deposition, whereas there was no IgA deposition in the glomeruli of the HKL group (Figure 2H-L). The histopathology of IgNA rats before and after drug treatment was scored according to the updated IgAN MEST-C scoring index. It was found that HKC or LEF could improve the kidney pathology of IgNA rats to a certain extent, and the effect seen in the HKL group was more obvious than that in the HKC or LEF group (Table 1). These results suggest that the use of HKL in the treatment of IgAN rats had a positive effect on the improvement of renal pathology.

### HKL inhibits the expression of the TGF-β1/Smad3 signaling pathway in IgAN rats

**Figure 3:**
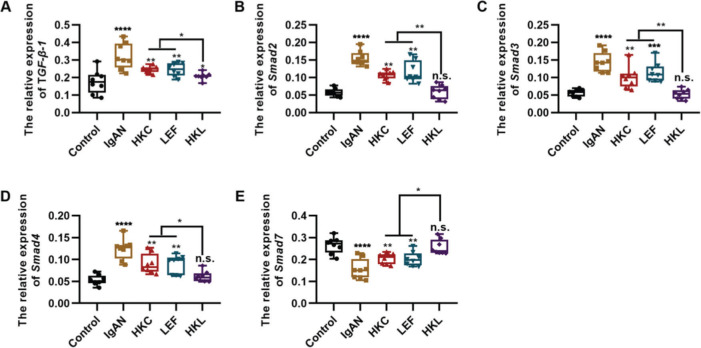
HKL inhibits the expression of the TGF-β1/Smad3 signaling pathway in IgAN rats. (A-D) Expression of TGF-β1 (A), Smad2 (B), Smad3, (C), and Smad4 (D) in rats of all groups; (E) HKL upregulates the expression of Smad7 in IgAN rats. HKC: Huangkui capsule, LEF: leflunomide, HKL: HKC in combination with LEF, IgAN: Immunoglobulin A nephropathy, *p<0.05, **p<0.001, ***p<0.0001, and ***p<0.0001, Kruskal-Wallis test followed by Dunn’s multiple comparisons test.

To determine the molecular mechanism of HKL in improving the pathogenesis of IgAN, we performed qRT-PCR and found that HKL downregulated the expression levels of TGF-β1, Smad2, Smad3, and Smad4 in IgAN rats and upregulated the expression levels of Smad7 (Figure 3A-E, *p<0.05, **p<0.001, ***p<0.0001, and ***p<0.0001). Therefore, we conclude that the TGF-β1/Smad3 signaling pathway may be involved in the ability of HKL to improve the health of IgAN rats.

### HKL inhibits the chemotaxis of Th22 cells

**Figure 4:**
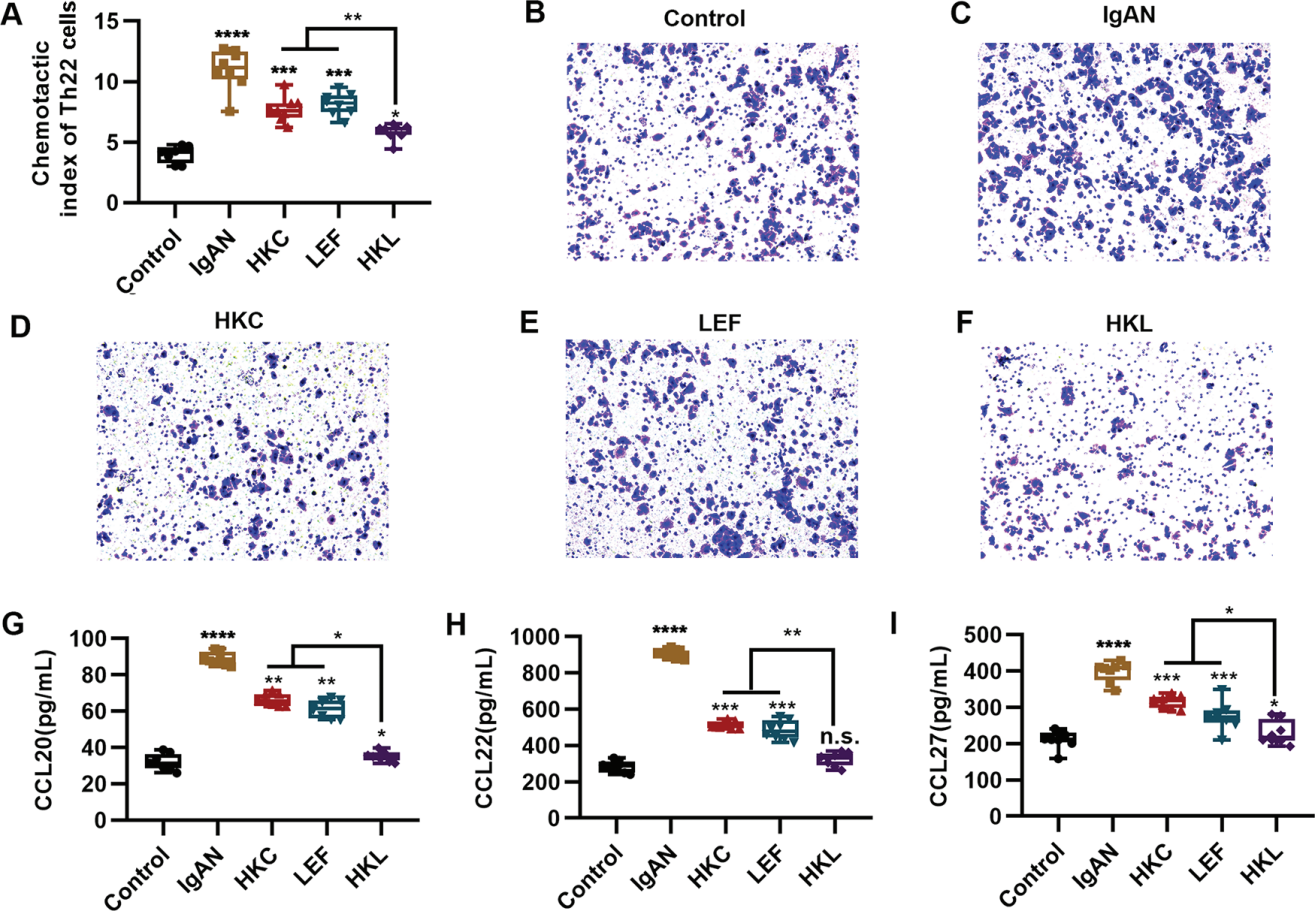
HKL inhibits the chemotaxis of Th22 cells. (A) Chemotaxis of Th22 cells in each group. (B-F) Detection of migrated Th22 cells via crystal violet staining. (G-I) ELISA analysis of the levels of CCL20 (G), CCL22 (H), and CCL27 (I) in each group. HKC: Huangkui capsule, LEF: leflunomide, HKL: HKC in combination with LEF, IgAN: Immunoglobulin A nephropathy, *p<0.05, **p<0.001, ***p<0.0001, and ***p<0.0001, Kruskal-Wallis test followed by Dunn’s multiple comparisons test.

An increased number of Th22 cells correlates with IgAN deterioration (25), which may be attributed to the infiltration and proliferation of lymphocytes (26), whereas lymphocyte migration in response to chemokine activities is the key to Th22 cell infiltration. The evaluation of the chemotaxis of Th22 cells in each group suggested that compared with the control group, Th22 cells in the IgAN group had a sharp increase in the chemotactic index. However, HKL treatment reversed the decrease in the chemotactic index of Th22 cells (Figure 4A, *p<0.05, **p<0.001, ***p<0.0001, and ***p<0.0001). Meanwhile, staining of the migrated cells demonstrated that HKL treatment could curb the migration ability of Th22 cells (Figure 4B-F). Existing data have shown that the chemotaxis of Th22 cells is regulated by chemotactic factors such as CCL20, CCL22, and CCL27 (25). To further validate the effect of HKL on CCL20, CCL22, and CCL27, we detected the levels of these indicators and found that they were overexpressed in the IgAN group, whereas HKL treatment reduced the secretion of CCL20, CCL22, and CCL27 (Figure 4G-I, *p<0.05, **p<0.001, ***p<0.0001, and ***p<0.0001).

### HKL inhibits the proliferation and activity of Th22 cells

**Figure 5:**
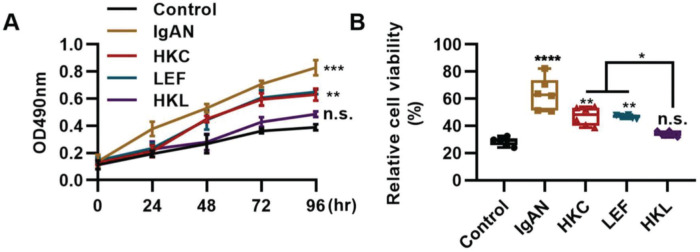
HKL inhibits the proliferation and activity of Th22 cells. (A) Results of the MTT assay show the change in the proliferation of Th22 cells in each group. (B) Results of the WST-1 assay show the changes in the viability of Th22 cells in each group. HKC: Huangkui capsule, LEF: leflunomide, HKL: HKC in combination with LEF, IgAN: Immunoglobulin A nephropathy, *p<0.05, **p<0.001, ***p<0.0001, and ***p<0.0001, Kruskal-Wallis test followed by Dunn’s multiple comparisons test.

The differentiation and proliferation of Th22 cells are conducive to the accumulation of Th22 cells. In this study, compared with the IgAN group, Th22 cells in the HKL group showed a sharp decrease in proliferation (Figure 5A, **p<0.001 and ***p<0.0001) and activity (Figure 5B, **p<0.001 and ***p<0.0001). With respect to the progression of IgAN, the migration of Th22 cells can further exacerbate the disease.

### Molecular mechanism underlying HKL-induced inhibition of the activity of Th22 cells

**Figure 6:**
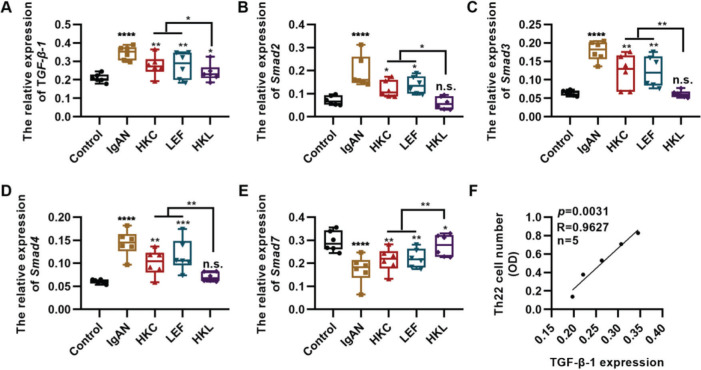
Molecular mechanism of HKL inhibiting the activity of Th22 cells. (A-D); qRT-PCR results reveal that HKL downregulates the expression of the TGF-β1/Smad3 signaling pathway. (E) HKL upregulates Smad7 expression. (F) The quantity of Th22 cells is positively correlated with TGF-β1 levels. HKC: Huangkui capsule, LEF: leflunomide, HKL: HKC in combination with LEF, IgAN: Immunoglobulin A nephropathy, *p<0.05, **p<0.001, ***p<0.0001, and ***p<0.0001, Kruskal-Wallis test followed by Dunn's multiple comparisons test.

TGF-β1 regulates proliferation and differentiation of Th22 cells (27). To further explore the *in vitro* molecular mechanism of HKL, we detected the *in vitro* expression of the key components of the TGF-β1/Smad3 signaling pathway in Th22 cells. Compared with the cells in the IgAN group, the expression levels of TGF-β1, Smad2, Smad3, and Smad4 were significantly inhibited, whereas those of Smad7 were upregulated in the cells of the HKL group (Figure 6A-E, *p<0.05, **p<0.001, ***p<0.0001, and ***p<0.0001). Furthermore, the number of Th22 cells was positively correlated with the level of TGF-β1 (Figure 6F).

## DISCUSSION

HKC and LEF are the most common drugs used for the treatment of inflammation, and existing data have revealed the molecular mechanisms of HKC and LEF for the treatment of lupus nephritis, chronic nephritis, and other immune diseases (13,16,28,29). However, there are insufficient data regarding the efficacy and molecular mechanism of HKL in the treatment of IgAN. In this study, we used IgAN rats and Th22 cells extracted from them to perform *in vivo* and *in vitro* experiments with the aim of uncovering evidence for deeper understanding of the efficacy and molecular mechanism of HKL in the treatment of IgAN.

Clinically, patients with IgAN mainly present with symptoms of microscopic or visible blood in the urine, proteinuria, hypertension, or kidney dysfunction (8). In this study, we found that the expression levels of the key components of the TGF-β1/Smad3 signaling pathway were upregulated in patients with IgAN and that IgAN rats after HKL treatment showed significant improvements in proteinuria and hematuria; the levels of protein and red blood cells in these rats were significantly lower than those in rats treated with a single drug. HE staining results also showed that HKL treatment significantly improved the glomerular pathological status of IgAN rats. Similarly, IHC staining demonstrated the effectiveness of HKL in IgAN rats. Therefore, HKL shows promise for the treatment of IgAN.

To identify the potential mechanism of HKL in the treatment of IgAN, we determined the expression profiles of some genes and found that HKL suppressed the activity of the TGF-β1/Smad3 signaling pathway in IgAN rats. Therefore, the mechanism of HKL in the treatment of IgAN may rely on the regulation of the TGF-β1/Smad3 signaling pathway.

Chronic inflammation is a common hallmark of IgAN (5,30). Therefore, we investigated the effect of HKL on IgAN in Th22 cells and demonstrated that HKL curbed the generation of TGF-β1 in Th22 cells. Interestingly, existing data have revealed a correlation between TGF-β1 and Th22 cells, similar to our findings (25,27). Th22 cells, a type of T helper cell, can activate other immune cells to regulate immunocompetence (31,32). Excessive proliferation of Th22 cells contributes to severe inflammation (20). Th22 cells can further exacerbate IgAN, particularly in patients with IgAN (26). CCL20, CCL22, and CCL27 are important for the functions of Th22 cells (26). The interaction between these chemotactic factors and Th22 cells can further induce lymphocyte anomalies in patients with IgAN (33,34). Studies have shown that infection and galactose-deficient IgA accumulation induces the secretion of chemotactic factors by glomerular membrane cells, thereby contributing to hyperplasia of the glomerular membrane and injury to mesangial cells (35,36). In this study, we found that HKL blocks the expression of CCL20, CCL22, and CCL27 in intercapillary cells, thereby mitigating the migration of Th22 cells.

**Figure 7:**
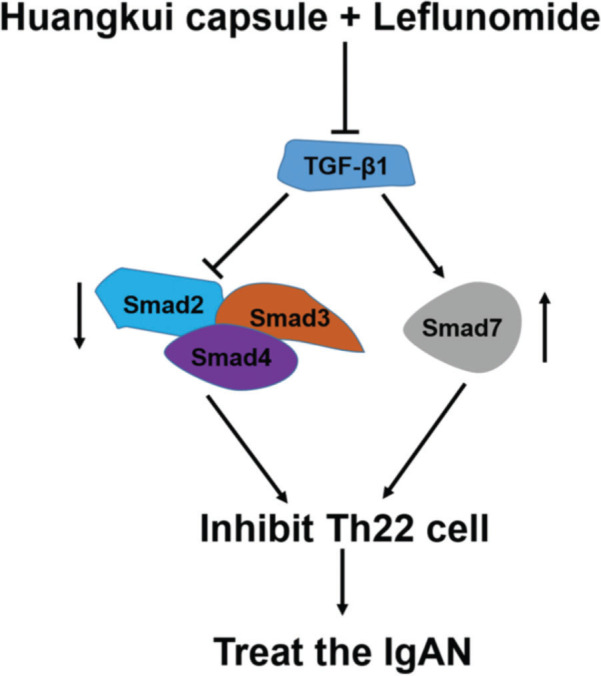
Functional model of HKL for the treatment of IgAN. TGF-β1, as a central regulatory factor, regulates the downstream expression of Smad2, Smad3, Smad4, and Smad7, thereby inhibiting the activity of Th22 cells. HKL: Huangkui capsule in combination with leflunomide, IgAN: Immunoglobulin A nephropathy.

To validate the accuracy of the above experiments, we evaluated the proliferation and viability of Th22 cells in patients with IgAN and found that HKL treatment decreased the proliferation and viability of Th22 cells, further confirming the above findings. These molecular findings suggest that HKL inhibits the activity of Th22 cells by regulating the TGF-β1/Smad3 signaling pathway, thereby ameliorating IgAN (Figure 7).

In conclusion, by targeting the TGF-β1/Smad3 signaling pathway, HKL can improve kidney injury in IgAN rats and can inhibit the excessive proliferation and metastasis of Th22 cells significantly better than treatment with a single drug.

## AUTHOR CONTRIBUTIONS

Shuwen Pei S wrote the manuscript. Pei S and Yan Li Y designed the study, collected the data and performed the statistical analysis. All of the authors reviewed and approved the final version of the manuscript.

## Figures and Tables

**Table 1:** Comparison of pathological examination results before and after drug treatment [n (%)].

MEST-C classification	Pretreatment of HKC	Post-treatment of HKC	Pretreatment of LEF	Post-treatment of LEF	Pretreatment of HKL	Post-treatment of HKL
M1	8	3*	8	4*	8	0***
E1	8	4*	8	2*	8	1***
S1	8	4*	8	4*	8	0***
T						
T0	0	0	0	0	0	0
T1	1	1	3	2	2	1
T2	7	2**	5	2*	6	0***
C						
C0	0	0	0	0	0	0
C1	2	0*	2	1	0	0
C2	6	3*	6	3*	8	0***

M: mesangial hypercellularity (M0<50%; M1⩾50% of the glomeruli), E: endocapillary hypercellularity (E0: absent; E1: present), S: segmental glomerulosclerosis (S0: absent; S1: present), T: tubular atrophy/interstitial fibrosis (T0: 0-24%; T1: 25-49%; and T2:⩾50% of the cortical area), C: cellular/fibrocellular crescents (C0: absent; C1: 1-24%; and C2:⩾25% of the glomeruli), HKC: Huangkui capsule, LEF: leflunomide, HKL: HKC in combination with LEF.

* *p*<0.05,

** *p*<0.01,

*** *p*<0.001.
